# Design, implementation, and evaluation of an innovative intelligence information management system for premature infants

**DOI:** 10.1177/20552076221127776

**Published:** 2022-10-11

**Authors:** Shahrbanoo Pahlevanynejad, Navid Danaei, Reza Safdari

**Affiliations:** 1 Department of Health Information Management, 48504 School of Allied Medical Sciences, 48439 Tehran University of Medical Sciences, Tehran, Iran; 2 Department of Health Information Technology, Sorkheh School of Allied Medical Sciences, 154203 Semnan University of Medical Sciences, Semnan, Iran; 3 Department of Pediatric, 154203 Semnan University of Medical Sciences, Semnan, Iran

**Keywords:** Premature infant, system evaluation, health information management, premature birth system, neonatal registry, prematurity

## Abstract

**Introduction:**

Low birth weight is the most important condition of neonatal community health and the main cause of neonates' mortality. Identifying the indexes associated with this condition, and factors to prevent, and managing related data can help reduce the birth of premature infants to reduce the mortality rate due to this condition. The goal of present study was to design, implement and evaluate an innovative intelligence information management system for premature infants.

**Material and method:**

The present study was a multidisciplinary research that was done in 2019 to 2021 in four integrated phases in Iran. The first phase aimed to compare the current status of registration systems of premature infants through a systematic review and semi-structured interviews by using the Delphi model Then the minimum data set was determined and was designed a proposed model based on it. In the second phase, the structure and how the user interacts with the system were determined, and, using Microsoft Visio software, Unified Modeling Language diagrams were drawn to define the logical relationship of data. In the third phase, the system was developed, and finally in the last phase, in three methods, users' views on the usability of the system were evaluated.

**Results:**

The findings of this study included 233 essential data elements that were placed in two main groups of essential data, and the system was approved by end users for 87.73% consent and 67.19% satisfaction for SUMI (Software Usability Measurement Inventory) and 7.97 of 9 in QUIS questionnaire.

**Conclusion:**

This research's results can be beneficial and functional such as a complete sample for design and development of other systems concerned to health systems.

## Introduction

Low birth weight is one of the most important indicators of community health and one of the main causes of infant mortality.^1,2^ About 120 million babies are born worldwide each year, about 25 million of whom are underweight at birth, and the proportion is about 50 percent in some parts of Asia.^3^ According to statistics reported each year, an average of 32 million low birth weight infants is born in low income countries, of which two-thirds of cases belong to Asia.^4^ The World Health Organization (WHO) has also reported a 10% prevalence of low birth weight in Iran.^5^ Due to the importance of the issue, November 17 has been named World Prematurity Day to raise awareness in the world.^6^ From an economic point of view, in addition to physical problems, low birth weight infants bring a lot of charges to the healthcare system of countries.^7^ It seems that identifying the factors associated with the disease, and ways to prevent them, and managing this data can help reduce the birth of premature infants and its complications and reduce the mortality rate due to this condition.^8^ This study aimed to design, implementation and evaluation of an innovative intelligence information management system for premature infants.

## Material and method

The present study was a descriptive, developmental study with an applied approach that was conducted in the years 2019 to 2021 in four stages in Semnan in IRAN. In the first stage, in order to identify and compare the current status of premature infant registration systems in all of the world, a systematic review in five major information databases containing PubMed, Scopus, Web of Science, IEEE, and Embase was done and then through three session of semi-structured interviews with using the Delphi model was accomplished until the MDS were determined and a proposed model was designed. In the second step, to determine the structure and how the user interacts with the system, UML diagrams were drawn using Microsoft Visio software to define the logical relationship of data, and user face was designed. In the third stage, the system was developed, and finally in the fourth stage, users' views on the usability of the system were evaluated in three methods.

## Results

**Figure 1.**
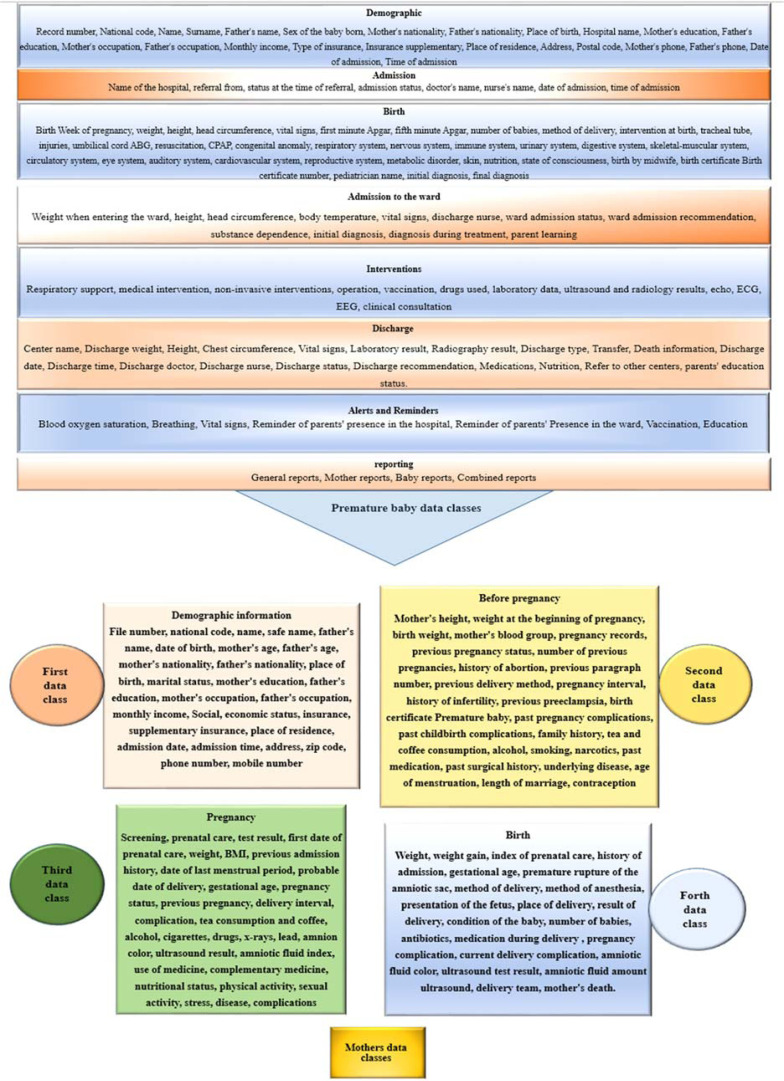
Design of premature infant registration system (separately for mother and infant data).

Findings from the first phase of this study included 233 essential data elements that were placed in two main groups of essential data: mother (107 elements) and infant (126 elements) shown in Figure 1.

**Figure 2.**
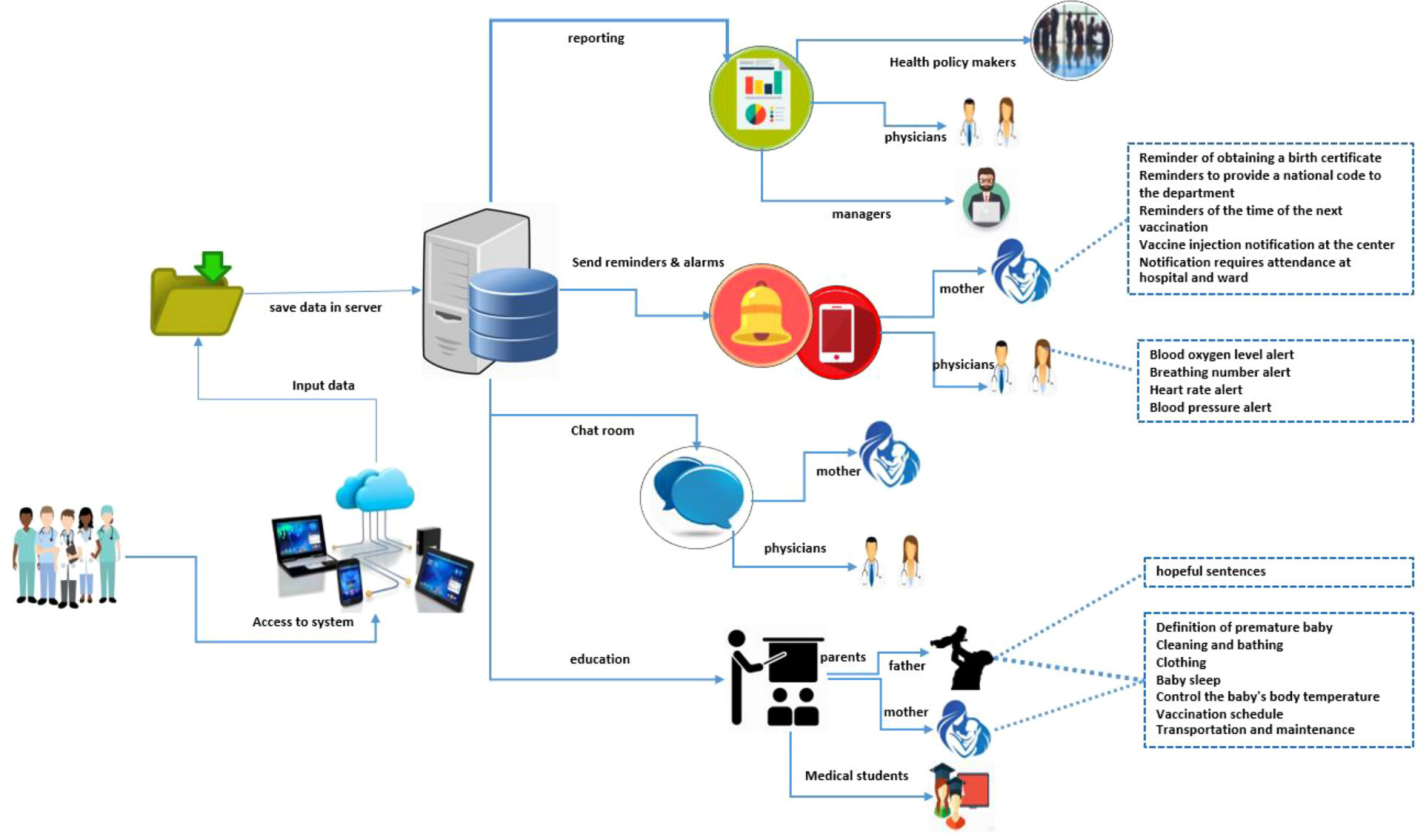
Premature infant information management system architecture.

The reliability of the questionnaire was obtained with Cronbach's alpha coefficient of 0.938 for the mother questionnaire and 0.915 for the infant questionnaire. In the second stage, the results of the study showed that the use of UML models and diagrams provides suitable conditions for flow modeling and information system development with a precise definition of the structure, system behavior, and how the user interacts with the system. The architecture of the conceptual model of a system and its final capabilities was shown in Figure 2.

The third step was to develop and evaluate a program using the C# programming language on the.NET 4.5 technology platform. Fourth-level results showed that the system was approved by end users with 87.73% consent and 67.19% satisfaction for SUMI (Software Usability Measurement Inventory) and 7.97 out of 9 in QUIS (User Interface Satisfaction Questionnaire), respectively.

## Conclusion

Due to the high prevalence of preterm birth in Iran and the great economic burden that is imposed on the family, society, and health systems; prevention, and disease management interventions are essential to improve the quality of neonatal health care. The results of this research can be useful and practical as a comprehensive model for the analysis, design, and development of all systems related to diseases and health systems.
